# The Application of Chemometrics to Volatile Compound Analysis for the Recognition of Specific Markers for Cultivar Differentiation of Greek Virgin Olive Oil Samples

**DOI:** 10.3390/foods9111672

**Published:** 2020-11-15

**Authors:** Ioanna S. Kosma, Michael G. Kontominas, Anastasia V. Badeka

**Affiliations:** Laboratory of Food Chemistry, Department of Chemistry, University of Ioannina, 45110 Ioannina, Greece; i.kosma@uoi.gr (I.S.K.); mkontomi@uoi.gr (M.G.K.)

**Keywords:** olive oil, volatile compound analysis, cultivar differentiation, markers, chemometrics

## Abstract

In the present study, volatile compound analysis of olive oil samples belonging to ten Greek cultivars was carried out. A total of 167 olive oil samples collected from two consecutive harvest years were analyzed by Head Space-Solid Phase Microextraction-Gas Chromatography/Mass Spectrometry (HS-SPME-GC/MS). Volatile compound data were combined with chemometric methods (Multivariate Analysis of Variance (MANOVA) and Linear Discriminant Analysis (LDA)) with the aim not only to differentiate olive oils but also to identify characteristic volatile compounds that would enable differentiation of botanical origin (marker compounds). The application of Stepwise LDA (SLDA) effectively reduced the large number of statistically significant volatile compounds involved in the differentiation process, and thus, led to a set of parameters, the majority of which belong to compounds that are highly dependent on variety. In addition, the use of these marker compounds resulted in an increased correct classification rate (85.6%) using the cross-validation method indicating the validity of the model developed despite the use of a large number of dependent variables (cultivars).

## 1. Introduction

Olive tree cultivation, one of the oldest and most important agricultural activities, has led to the diversification of olives into a large number of cultivars. The olive fruit cultivar is a major determinant of olive oil quality due to differences in specific aroma and taste, phenolic content, etc. leading to a variety of olive oils, each with unique flavor characteristics and stability. In Greece more than 30 cultivars of olives exist, and most are characteristic of cultivation area (i.e., Galano cultivar from Metagitsi of Halkidiki, Topiki Makris cultivar from Evros, Samothrakis cultivar from Samothraki island, etc.) [1,2].

According to the International Food Authenticity Assurance Organization (ΙFAAO) [3], “food authenticity is the process of irrefutably proving that a food or food ingredient is in its original, genuine, verifiable and intended form as declared and represented”. Food authentication is the concern of (i) regulatory authorities to avoid food adulteration (ii) food processors that do not wish to be subjected to unfair competition from unscrupulous processors who would gain an economic advantage from the misrepresentation of the food they are selling and (iii) the rights of consumers who expect to purchase and consume genuine, unadulterated and quality foods for which they usually pay a premium price. Having all this in mind, the European Union has issued regulation 178/2002 [4] regarding the quality, safety and traceability of commercially available foods.

Sophisticated analytical techniques used for the authentication of olive oil include: analysis of volatile compounds by the HS-SPME-GC/MS method [1,2], proton transfer mass spectrometry (PTR/MS) [5], isotopic ratio mass spectroscopy (IRMS) [6,7], nuclear magnetic resonance (NMR) [8,9,10], DNA molecular techniques [11,12,13], two dimensional gas chromatography (GC x GC) [14], liquid chromatography with DAD or mass spectrometry [15,16], inductively coupled plasma spectroscopy (ICP) [17], etc. 

Olive oil is generally defined on the basis of its sensory as well as its physico-chemical characteristics. Within sensory evaluation, many volatile compounds are considered responsible for desirable and undesirable odor attributes of olive oil. As volatiles are influenced by many factors (olive cultivar, fruit ripeness, local climatic and soil conditions, specific agriculture practices, processing and packaging conditions, etc.) [18], variation is observed in the olive oil aroma profile. A high concentration of volatile compounds in olive oil does not guarantee the intensity of its aroma. This can be explained by the fact that the aromatic profile may include compounds with a very low olfactory threshold, in contrast to others that are present in higher concentrations and yet, contribute very little or not at all to olive oil aroma. Either major or minor volatile compounds are crucial for olive oil quality. The wide variety of volatile compounds present in high quality virgin olive oil belong to several chemical groups. The most important groups are the C5 and C6 compounds that include aldehydes, alcohols, esters and hydrocarbons. These compounds are produced via the lipoxygenase pathway (LOX) and fatty acid and amino acid metabolism [18,19,20]. 

The sensory evaluation protocol for olive oil, namely, the Panel Test, has been established by the International Olive Oil Council [21] and EU regulations [22,23]. In this procedure, a group of more than eight trained panelists detects the presence of different sensory attributes in virgin olive oil samples and scores their intensity into a scale from 0 to 10. Several disadvantages of this evaluation technique, however, include the lack of reference standards, low repeatability of data, and to a certain degree subjectivity, etc. [24]. The volatile profile of an olive oil sample represents a fingerprint of the specific sample and gas chromatography (GC) coupled to mass spectrometry (MS) has been the most widely employed technique in combination with SPME that has been successfully applied for the classification of olive oils according to cultivar and/or geographical origin [1,2,25,26,27,28,29]. 

Previously published data on the differentiation of olive oil cultivars by means of instrumental analysis combined with chemometrics have led to the conclusion that this combination is a very powerful tool for the determination of authenticity of olive oil [1,2,25]. Literature data has shown that volatile compound analysis *per se* or in combination with other analytical parameters resulted in a greater aggregation of olive oil samples in respective cultivars. This fact and the potential of this analytical technique have been used in the present study, the aim of which was to determine those volatiles which have a higher discriminant ability for cultivar differentiation of olive oil. For this purpose, the volatile compound data from ten different olive oil cultivars were combined and analyzed using Multivariate Analysis of Variance (MANOVA) and Linear Discriminant Analysis (LDA). As the number of significant variables (*p* < 0.05) that derive from MANOVA may be quite large, stepwise LDA (SLDA) was then applied in order to reduce this number of volatiles to that which could be considered as the best set of predictors-markers of authenticity in relation to the herein studied cultivars. 

## 2. Materials and Methods 

### 2.1. Olive Oil Samples

A total of 167 virgin olive oil samples were collected during the harvest years 2012–2013 and 2013–2014 from various regions in Greece belonging to ten different cultivars, namely: *Ladolia Kerkyras*, *Galano*, *Adramitiani*, *Samothraki*, *Athinolia*, *Hontrolia*, *Koutsourelia*, *Kolovi*, *Topiki Makris* and *Manaki* (Table 1). Sampling was carried out during the months November until the end of January of each year. Olives were picked by hand at the stage of optimum maturity (maturity index 5–6). All olive samples were washed, crushed and fed to the malaxation station where the olive paste was processed at 30 °C for a period of 30 min. The paste was then separated by centrifugation at 35 °C. According to Guerrini et al. [30] an increase in temperature between malaxation and centrifugation positively affects olive oil quality and yield. Finally, olive oil was filtered and stored in dark glass bottles at 4 °C until analysis. 

### 2.2. Determination of Free Acidity, Peroxide Value and Absorption Coefficients (K_232_, K_270_)

The conventional quality parameters were determined according to the Official EU method [22]. All determinations were carried out in triplicate. 

### 2.3. HS-SPME-GC/MS

The determination of volatile compounds was carried out according Kosma et al. [1], using SPME in combination with GC/MS. Semi-quantification of volatile compounds was carried out using the internal standard method. Concentrations were calculated using the following formula:

$$C_{x}=\frac{AREA_{x}\times C_{i}}{AREA_{i}}$$

where *C_x_* = concentration of the unknown compound, *C_i_* = concentration of the internal standard solution, *AREA_x_* = peak area of the unknown compound and *AREA_i_* = peak area of the internal standard solution); results were expressed as μg/kg. All determinations were carried out in triplicate.

### 2.4. Statistical Analysis

Statistical treatment of data was performed using SPSS 25.0 software. Data were subjected to MANOVA in order to determine those variables that are significant for the differentiation of olive oil cultivar. Cultivar was taken as the independent variable, while volatile compounds were taken as the dependent variables. Pillai’s Trace and Wilks’ Lambda indices were computed to determine a possible significant effect of experimental parameter values on olive oil cultivar. LDA was then applied using the selected dependent variables in order to explore the potential for classification of olive oil samples according to cultivar. Original and leave-one-out cross-validation methods were used to test the prediction classification ability. In the original method, the prediction rate results from the contribution of all cases in the discriminant functions while in cross-validation, a randomly chosen parameter, is classified in a group based on a discriminant function, created by all the other parameters (except the randomly chosen one). This procedure is repeated for all the parameters of the tested sample. The homogeneity of variability was tested by application of the Box M index [31]. 

Since the number of volatile compounds resulting from the analysis is quite large, SLDA was used as a final step in order to determine those variables that show higher discriminant ability. The SLDA method is based on the creation of an initial model which does not include any of the significant variables-predictors. The predictors are introduced in the analysis sequentially, one-at-a-time, until all are included in the model. The SLDA classification method applies a forward variable selection algorithm using Wilks’ Lambda as a selection criterion and the F-statistical factor in order to determine the significance of changes in Wilks’ Lambda when the impact of a new variable is evaluated [32]. Before a new variable enters the classification model, the step-by-step process checks if all previous variables remain significant. If any of these are no longer significant, they are removed from the model and the process continues until there are no other variables that meet the entering standard or when the variable that will be inserted next is the one that was just rejected; at this point the variable selection process stops [33]. Thus, the SLDA procedure is guided by the corresponding F-to enter and the F-to remove values. The F-value, for a variable, indicates its statistical significance in discriminating between groups which is a measure of the degree a variable contributes to predicting group membership. The criteria for entry and removal are set by default, given by the statistical software, i.e., minimum F to enter the analysis is 3.84, maximum F to remove from the analysis is 2.71 [34]. The evaluation of the SLDA classification results was conducted using the leave-one-out method. 

## 3. Results

### 3.1. Analysis of Conventional Quality Parameters 

As shown in Table 2 the majority of olive oil samples tested were categorized as extra virgin olive oil since their acidity, peroxide value and absorption coefficients (K_232_ and K_270_) did not exceed the internationally established limits set by the EU Regulation [22]. Specifically, acidity recorded values between 0.3% ± 0.2 in *Galano* samples to 1.8% ± 1.6 in *Adramitiani* samples which along with samples from *Ladolia Kerkyras*, Samothraki and *Athinolia* (1.3 ± 1.5, 0.9 ± 0.6 and 0.8 ± 0.6, respectively) recorded higher acidity values and were categorized as virgin olive oil. Furthermore, *Ladolia Kerkyras* recorded the highest K_232_ value (2.76 ± 1.02) this categorizing this oil as “lampante” while the other samples remained lower than the internationally established limit of 2.50 [22]. 

In a similar study by Pouliarekou et al. [35] who classified olive oil samples from Western Greece according to cultivar and geographical origin, high values of quality parameters for samples belonging to *Lanolia Kerkyras* were also recorded. These samples were also categorized as “lampante” and according to the authors this may be related to the method of olive fruit collection, where in certain olive orchards in Kerkyra, fruits are left to fall off the olive tree and are collected in nets on the ground. In general, this collecting method is not a common practice as it causes damages to the fruit.

### 3.2. Analysis Volatile Compound Analysis

Sixty volatile compounds were identified and semi-quantified in olive oil samples tested (Table 3). These volatiles included alcohols, aldehydes, ketones, esters and hydrocarbons. The higher total concentration was recorded for the cultivars *Ladolia Kerkyras* (47,765.9 μg/kg), *Topiki Makris* (43,172.2 μg/kg) and *Hontrolia* (42,132.9 μg/kg).

The lipoxygenase pathway has a major contribution to virgin olive oil aroma as a wide variety of volatile compounds are produced through this biological pathway [19]. Aldehydes represented the most abundant chemical class, being the products of the lipoxygenase pathway, which starts right after damage of olive fruit tissues due to the release of enzymes that oxidize and cleave polyunsaturated fatty acids. *(E)*-2-Hexenal was the most abundant aldehyde identified in all samples tested, related to olive fruit maturity (characteristic of olive cultivar) and oxidation stage of olive oil [20,35] recording its higher concentration in the *Topiki Makris* cultivar (27,638.6 ± 4366.2 μg/kg). Hexanal followed, recording its highest concentration in the *Ladolia Kerkyras* (3774.7 ± 2983.8 μg/kg) and *Topiki Makris* (3189.8 ± 1057.7 μg/kg) samples. It should be noted that the relatively high hexanal content observed in the olive oil samples tested does not necessarily indicate either oxidized olive oils or olive oils in the early stages of oxidation. According to Morales et al. [36], and Vichi et al. [37], hexanal levels cannot distinguish oxidized from “virgin” olive oils as they come from both the lipoxygenase pathway and chemical oxidation of olive oil. (*E*)-2-Pentenal derives from the lipoxygenase pathway through the action of alkoxy radicals on linolenic acid 13-hydroperoxides producing the corresponding alcohol (*(Z)*-2-pentenol) which is subsequently oxidized. *(E)*-2-pentenal was not detected in the *Adramitiani*, *Samothrakis*, *Athinolia*, *Ladolia Kerkyras* and *Manaki* cultivars, while it showed the highest concentration in the *Topiki Makris* cultivar (67.7 ± 55.5 μg/kg). According to Morales et al. [36], and Kiritsakis [38], pentanal, octanal, nonanal and hexanal are the main compounds that form in oxidized olive oils, whereas in the samples tested, the first three were found at relatively low levels. Of these, pentanal and nonanal were identified in all olive oil samples, while octanal was not detected in the in *Manaki* cultivar. 

Aldehydes are reduced to alcohols through the action of alcohol dehydrogenase. *(E)*-2-Hexenol was the most abundant alcohol recording its highest concentration in the *Koutsourelia* (1397.7 ± 1385.0 μg/kg), while hexanol, the second most abundant alcohol recorded the highest concentration in the *Kolovi* (1704.6 ± 1079.5 μg/kg) samples. These two alcohols can be used for cultivar differentiation while (*E*)-2-hexenol is responsible for the characteristic “green” aroma notes of the olive oil; hexanol’s odor perception is considered as fruity, banana like and grassy [1,2]. 1-Penten-3-ol, deriving from the lipoxygenase pathway through the action of alkoxy radicals on 13-hydroxy peroxides of linolenic acid [18], was present in all olive oil samples and its concentration ranged from 6.5 ± 23.5 μg/kg in the *Samothrakis* samples to 165.5 ± 125.4 μg/kg in *Koutsourelia*.

Another important chemical class that has a major contribution to olive oil aroma is that of esters. Esters derive from the lipoxygenase pathway, through the action of alcohol acyltransferase that catalyzes the formation of acetate esters through acetyl-CoA derivatives. Despite the fact that esters comprise minor components of olive oil aroma their contribution is quite significant as they complement aroma with sweet and pleasant notes [18,39]. *Kolovi* (3307.1 μg/kg), *Manaki* (2870.6 μg/kg) and *Koutsourelia* (2710.4 μg/kg) samples recorded the highest total concentrations compared to the other cultivars while esters were not identified in the volatile fraction of the *Galano* and *Hontrolia* olive oils. The absence of esters in the volatile fraction of certain cultivars may be due to the action of the enzyme alcohol acyltransferase. The activity of this enzyme is significantly influenced by pH (6.8–8) and temperature (35 °C), as well as the availability of the appropriate substrate. The activity of alcohol acyltransferase can be enhanced by cultivar selection as well as by modifying olive oil extraction conditions, i.e., operation at lower temperatures to prevent inactivation of the enzymes and promotion of esterification reactions [39]. 

Regarding ketones, their highest total concentration was recorded in the *Hontrolia* (3630.0 μg/kg) and *Ladolia Kerkyras* (3570.2 μg/kg) while *Samothrakis* recorded the lowest concentration (206.0 μg/kg). 1-Penten-3-one also derives from the lipoxygenase pathway and is positively correlated to bitter taste [39]. In olive oil samples tested, the lowest and highest concentration of 1-penten-3-one appeared in the *Samothrakis* and *Topiki Makris* cultivars (9.1 ± 22.4 μg/kg and 284.3 ± 148.8 μg/kg, respectively). 2-Pentanone and 3-pentanone resulting from homolytic cleavage processes, are also responsible for the green notes of aroma [40]. The highest concentrations of 2-pentanone and 3-pentanone occurred in the *Kolovi* cultivar (69.0 ± 130.0 μg/kg and 410.4 ± 271.3 μg/kg, respectively). Furthermore, 6-methyl-5-hepten-2-one was present in all samples tested with the exception of the *Manaki* samples. This ketone is produced through the action of the pseudomonads that break down terpene alcohols present in olive oil [41]. The organoleptic perception of this compound is characterized as pleasant, green, fruity and spicy, but when it exceeds the odor threshold (1.0 mg/kg) it gives an unpleasant aroma [41,42]. The 6-Methyl-5-hepten-2-one showed the highest concentration in *Adramitiani* cultivar (260.6 ± 240.3 μg/kg). 

In the intact tissues of the olive fruit there are only a few volatile components, mainly hydrocarbons which do not contribute to fruit aroma. Most of the volatile compounds form as soon as the tissues are damaged and the various enzymatic activities, previously mentioned, initiate [43]. Significant variations were observed in the total amount of hydrocarbons as these were found at low concentrations in most of the samples with the exception of *Ladolia Kerkyras* (13006.4 μg/kg) and *Hondrolia* (15966.5 μg/kg). The lowest total amount of hydrocarbons was recorded in the *Kolovi* cultivar (1351.1 μg/kg). 3-Ethyl-1,5-octadiene deriving from the lipoxygenase pathway, recorded significant variations in all samples tested, indicating the influence of cultivar and/or environmental conditions on olive tree growing. Specifically, the highest value of 3-ethyl-1,5-octadiene was recorded in the *Topiki Makris* (858.9 ± 343.2 μg/kg) and the lowest in *Athinolia* (198.7 ± 148.4 μg/kg).

According to Bubola et al. [44], some volatile terpenes have been suggested as useful indicators for the geographical and botanical differentiation of virgin olive oil. The total amount of terpenes showed significant variations among cultivars. Three cultivars appeared to have a fairly high content of terpenes namely, *Ladolia Kerkyras* (3781.4 μg/kg), *Adramitiani* (5476.3 μg/kg) and *Kolovi* (4405.3 μg/kg). The terpenes identified and semi-quantified were α-pinene, δ-3-carene, p-cymene, dl-limonene, (*E*)-β-ocimene, allo-ocimene, α-copaene, (*E*)-β-farnesene, (*E,E*)-α-farnesene and α-muurolene.

### 3.3. Multivariate Analysis of Variance

As a first step, the 167 olive oil samples were subjected to MANOVA in order to determine those volatile compounds which are significant for cultivar differentiation. Dependent variables included the total 60 volatile compounds identified and semi-quantified while cultivar was taken as the independent variable. Pillai’s Trace = 7.049 (F = 6.059, *p* = 0.001 < 0.05) and Wilks’ Lambda = 0.001 (F = 8.875, *p* = 0.001 < 0.05) index values showed the existence of a significant multivariable effect of cultivar on the identity of volatile compounds.

### 3.4. Linear Discriminant Analysis

**Figure 1:**
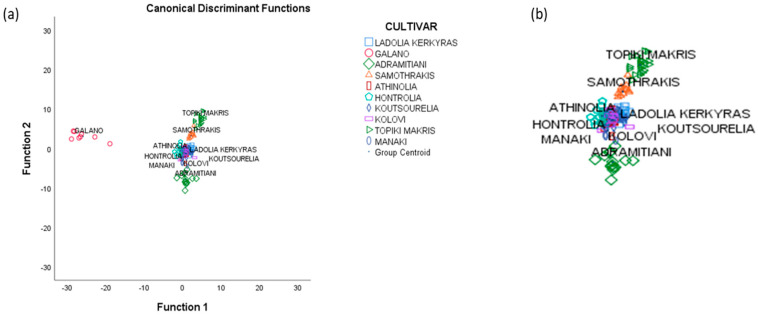
(**a**) Olive oil cultivar differentiation based on volatile compound analysis, distribution diagram obtained after the application of LDA (99.4% original, 83.2% cross-validation), (**b**) blow up of Figure 1a.

Fifty-five volatiles were found to be significant (*p* < 0.05) for cultivar differentiation and thus, were subjected to LDA, a second step of the analysis. Results showed that three statistically significant discriminant functions are formed (Table 4). A significant value of Wilks’ Lambda index shows that the discriminant function is basic for the differentiation of the investigated groups. Testing of the uniformity of variability (Box M index = 736.955, F = 3.134, *p* = 0.050) was insignificant at the 95% confidence level indicating the existence of uniformity of sample variability for each cultivar. In Figure 1a it is shown that olive oil samples from *Galano* are very well differentiated from the other cultivars. In Figure 1b it is clear that *Samothraki*, *Topiki Makris* and *Adramitiani* cultivars are well differentiated while the other cultivars are overlapping. The overall correct classification rate was 99.4% for the original and 83.2% for the cross-validation method. Correct cultivar classification (100%) was achieved for *Adramitiani*, *Samothraki* and *Galano* cultivars. 

Regarding five of the above olive cultivars (i.e., *Galano*, *Samothrakis*, *Adramitiani*, *Athinolia* and *Ladolia Kerkyras*) previously published work [1] showed that the application of LDA to olive oil volatiles led to a very satisfactory classification rate (97% original, 83% cross-validation), while *Galano* and *Samothraki* cultivars were fully differentiated from the rest of the cultivars investigated. Furthermore, regarding the other five cultivars (i.e., *Hontrolia*, *Koutsourelia*, *Kolovi*, *Topiki Makris* and *Manaki*) the classification rate achieved in previous work was 100% for the original and 82.4% for the cross-validation method also leading to very satisfactory differentiation of the tested cultivars [2]. In the present study, despite the quite large number of cultivars and dependent variables, the statistical model used showed a promising potential for olive oil cultivar differentiation. Combining the volatile compound analysis data of the ten cultivars, the classification rate slightly increased to 83.2% while four of the ten cultivars (*Galano*, *Samothrakis*, *Topiki Makris* and *Adramitiani*) were well differentiated.

### 3.5. Stepwise Linear Discriminant Analysis

**Figure 2:**
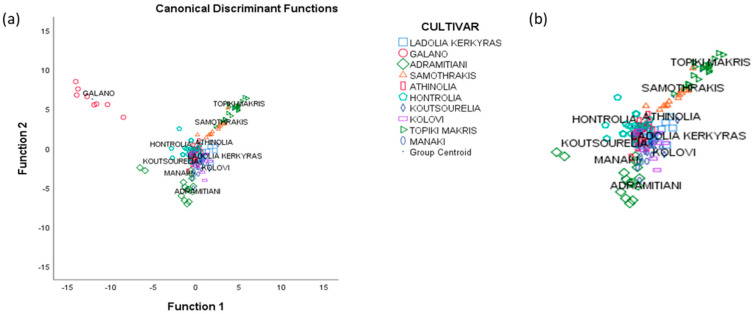
(**a**) Olive oil cultivar differentiation based on volatile compound analysis, distribution diagram obtained after the application of SLDA (93.4%original, 85.6% cross-validation), (**b**) blow up of Figure 2a.

As the final step of data statistical treatment, SLDA was used in order to select the variables with the higher discriminant ability. Of the 55 significant volatile compounds only 17 were found to have a higher discriminant ability (Table 5). Three statistically significant discriminant functions were formed (Table 4). As shown in Figure 2a, the *Galano* samples are very well differentiated. Figure 2b shows that olive oil samples from *Samothraki*, *Topiki Makris* and *Adramitiani* cultivars are also adequately differentiated while all other cultivars are overlapping. The overall correct classification rate was 94% for the original and 85.6% for the cross-validation method, somewhat increased in this case. Correct cultivar classification (100%) was achieved only for *Galano*, *Adramitiani* and *Topiki Makris* cultivars. 

Of the 17 volatile compound-markers, 5 derive through the lipoxygenase pathway [1-penten-3-ol, *(E)*-2-hexen-1-ol, *(E)*-2-pentenal, *(E)*-2-hexenal and hexyl acetate. This fact indicates strong dependence of this biological pathway on cultivar as these compounds have a major contribution to virgin olive oil aroma with a wide variety of volatile compounds being produced through this biological pathway [13]. Pizarro et al. [32], in an attempt to recognize volatile markers for the geographical discrimination of Spanish olive oil samples, identified six volatile compounds as markers, all deriving from the lipoxygenase pathway. According to Angerosa et al. [18] the effect of cultivar can be demonstrated by the various amounts of C6 compounds resulting from the LOX pathway for oils obtained under the same operating conditions collected at the same ripening stage. Furthermore, minor dependence of the number of volatiles from climatic conditions and the geographical area of cultivation emphasizes that cultivar is the dominant factor affecting the aroma formation of olive oil. This feature, in combination with the different concentration of (*E*)-2-hexenal, represents an effective tool for differentiating single-variety oils from different varieties. Focusing on specific volatile compound-markers, Kesen et al. [42] reported that the most abundant aldehyde was (*E*)-2-hexenal, followed by hexanal, in Turkish olive oil samples belonging to the Halhali cultivar. Furthermore, Bubola et al. [44] reported that among the 50 volatile compounds identified in olive oils from Bova cultivar, C6 compound (*E*)-2-hexenal was the most abundant aldehyde, while Tanouti et al. [40] recorded that the main volatile compounds present in olive oil samples produced in eastern Morocco were C6 compounds such as hexanal, (*E*)-hex-2-enal, Z-3-hexen-1-ol and 1-hexanol, as in the present study. Finally, Issaoui et al. [26] who studied the effect of the growing area of cultivation on aroma profiles of Chemlali and Chetoui cultivars, recorded that (*E*)-2-hexenal and 1-hexanol can be used as potential indicators, in this case for geographical differentiation. Ethanol, according to Kalua et al. [39], results from the fermentation process that takes place in the olive fruit before oil extraction contributing wine aroma notes to olive oil. It, thus, can be considered as a sugar fermentation marker. Nonanal is one of the main compounds that forms in oxidized olive oils and is associated with oil sensory defects [36,38,41]. Thus, nonanal can be considered as a possible marker of early oxidation processes in olive oil. Finally, terpene concentration significantly varies depending on cultivar and geographical origin and terpenes have been suggested as indicators for virgin olive oil differentiation [42,44]. In the present study three terpenes (dl-limonene, (*E*)-β-ocimene and α-copaene) were identified as volatile markers. According to Zunin et al. [45], α-copaene along with α-muurolene and α-farnesene were the terpenes that aided the discrimination between extra virgin olive oil from West Liguria from those of other Mediterranean regions. 

## 4. Conclusions

In the present study, volatile compound analysis from olive oil samples belonging to ten different cultivars from Greece was carried out in an effort to (i) to differentiate cultivar based on volatile compounds and (ii) to investigate the selection of potential markers leading to a successful cultivar differentiation. The results of the statistical treatment (MANOVA/LDA) showed that the differentiation of olive oil samples according to cultivar is possible despite the quite large number of cultivars investigated (83.2% cross-validation). Furthermore, the results obtained after the application of SLDA to the selected set of variables (55 volatile compounds) led to a quite reduced set of data (17 volatile compounds). These compounds provide a higher discriminant ability compared to the other volatiles, increasing the classification rate to 85.6% with the application of the cross-validation method. The specific volatile compounds identified as markers included a total of seventeen compounds. Of these, five derive from the lipoxygenase pathway (hexyl acetate, (*E*)-2-hexenal, (*E*)-2-hexenol, 1-penten-3-ol, (*E*)-2-pentenal) while three are terpenes (α-copaene, *(E)*-β-ocimene, dl-limonene), indicating a strong dependence of cultivar on the formation of olive oil’s volatile fraction. The resulting distribution diagram showed that the cultivars *Galano*, *Samothrakis*, *Topiki Makris* and *Adramitiani* were clearly differentiated. The results are quite encouraging, demonstrating the validity of the statistical model developed for the authentication of olive oil in relation to the differentiation of olive cultivars.

## Figures and Tables

**Table 1:** Olive oil samples collected.

Cultivars (Origin)	Number of Samples
*Topiki Makris* (Evros)	21
*Samothraki* (Samothraki isle)	13
*Galano* (Metagitsi-Chalkidiki)	8
*Hontrolia* (Chalikidiki)	21
*Adramitiani* (Lesvos isle)	15
*Kolovi* (Lesvos isle)	8
*Ladolia Kerkyras* (Corfu isle)	21
*Koutsourelia* (Etoloakarnania)	18
*Manaki* (Korinthos)	6
*Athinolia* (Lakonia)	36

**Table 2:** Conventional Quality Parameters.

Cultivars	% Acidity	PV (meq O_2_/kg)	Κ_232_	Κ_270_
*Topiki Makris*	0.6 ± 0.3 ^a^	12.3 ± 3.6 ^ab^	2.38 ± 0.38 ^bc^	0.15 ± 0.03 ^ab^
*Samothraki*	0.9 ± 0.6 ^ab^	8.7 ± 2.9 ^a^	1.89 ± 0.31 ^ab^	0.20 ± 0.03 ^b^
*Galano*	0.3 ± 0.2 ^a^	10.7 ± 2.6 ^a^	1.97 ± 0.21 ^ab^	0.14 ± 0.03 ^a^
*Hontrolia*	0.4 ± 0.2 ^a^	9.0 ± 3.2 ^a^	1.77 ± 0.27 ^a^	0.15 ± 0.03 ^a^
*Adramitiani*	1.8 ± 1.6 ^c^	11.2 ± 7.4 ^ab^	2.39 ± 0.86 ^bc^	0.17 ± 0.05 ^ab^
*Kolovi*	0.4 ± 0.2 ^a^	10.2 ± 5.0 ^a^	2.04 ± 0.45 ^ab^	0.16 ± 0.04 ^ab^
*Ladolia Kerkyras*	1.3 ± 1.5 ^bc^	15.6 ± 8.7 ^b^	2.76 ± 1.02 ^c^	0.20 ± 0.06 ^b^
*Koutsourelia*	0.5 ± 0.4 ^a^	10.0 ± 4.5 ^a^	2.04 ± 0.82 ^ab^	0.17 ± 0.04 ^ab^
*Manaki*	0.4 ± 0.2 ^a^	7.8 ± 1.2 ^a^	1.68 ± 0.22 ^a^	0.13 ± 0.03 ^a^
*Athinolia*	0.8 ± 0.6 ^ab^	10.8 ± 5.2 ^a^	1.93 ± 0.25 ^ab^	0.13 ± 0.02 ^a^

Means with different letters in the same row are significantly different (*p* < 0.05).

**Table 3:** Mean values and standard deviation of volatile compounds (μg/kg) identified in olive oil samples.

	Topiki Makris	Samothrakis	Galano	Hontrolia	Adramitiani	Kolovi	Ladolia Kerkyras	Koutsourelia	Manaki	Athinolia	RI_lit_^1^	RI_exp_^2^
**Alcohols**												
Ethanol	22.8 ± 104.4 ^a^	83.9 ± 205.4 ^a^	238.9 ± 258.5 ^a^	153.8 ± 300.9 ^a^	1348.4 ± 1117.8 ^b^	2282.2 ± 2386.9 ^c^	104.5 ± 412.7 ^a^	172.9 ± 244.5 ^a^	1323.7 ± 682.0 ^b^	286.6 ± 453.3 ^a^	<500	-^3^
1-Penten-3-ol	87.3 ± 46.2 ^b^	6.5 ± 23.5 ^a^	77.9 ± 51.6 ^b^	40.8 ± 65.8 ^ab^	64.2 ± 50.6 ^ab^	103.1 ± 72.6 ^b^	73.3 ± 77.9 ^b^	165.5 ± 125.4 ^c^	83.5 ± 44.7 ^b^	66.7 ± 65.3 ^b^	682	686
1-Pentanol				3.3 ± 15.0 ^a^	31.5 ± 69.3 ^ab^	52.2 ± 62.4 ^b^	17.3 ± 59.6 ^ab^	62.4 ± 131.0 ^b^	16.5 ± 40.4 ^ab^		768	766
(Z)-2-Pentenol	25.9 ± 48.0 ^a^			6.6 ± 30.4 ^a^	6.3 ± 16.8 ^a^	101.7 ± 86.5 ^b^	25.6 ± 50.2 ^a^	100.4 ± 116.9 ^b^		14.1 ± 36.4 ^a^	770	767
(E)-2-Hexenol	855.7 ± 584.6 ^ab^	1387.1 ± 844.2^b^	519.6 ± 466.5 ^a^	1336.0 ± 1068.0 ^b^	505.6 ± 457.8 ^a^	419.2 ± 278.4 ^a^	829.5 ± 551.5 ^ab^	1397.7 ± 1385.0^b^	632.0 ± 321.9 ^a^	327.3 ± 220.6 ^a^	867	862
Hexanol	655.0 ± 378.5 ^ab^	1222.4 ± 1064.1^cd^	325.4 ± 339.3 ^a^	637.5 ± 684.1 ^ab^	603.0 ± 525.3 ^ab^	1704.6 ± 1079.5 ^cd^	460.8 ± 778.3 ^ab^	935.7 ± 918.4 ^bc^	1335.0 ± 658.3 ^d^	320.9 ± 230.3 ^a^	870	862
**Total Alcohols**	**1646.7**	**2699.9**	**1161.9**	**2178.0**	**2600.6**	**4663.1**	**1511.1**	**2834.5**	**3390.6**	**1027.2**		
**Aldehydes**												
Butanal, 3-methyl-				1039.8 ± 4765.0 ^a^	6.9 ± 18.9 ^a^	3.4 ± 9.7 ^a^	15.3 ± 34.8 ^a^	39.4 ± 69.5 ^a^		7.7 ± 20.3 ^a^	656	660
Pentanal	151.7 ± 171.8 ^b^	155.2 ± 89.4 ^b^	68.8 ± 121.9 ^ab^	9.2 ± 42.1 ^a^	141.7 ± 161.2 ^ab^	36.0 ± 70.1 ^ab^	122.0 ± 315. 7 ^ab^	71.7 ± 84.2 ^ab^	7.8 ± 19.2 ^a^	16.5 ± 43.9 ^a^	699	695
(E)-2-Pentenal	67.7 ± 55.5 ^b^		10.0 ± 9.0 ^a^	7.6 ± 34.7 ^a^		7.7 ± 21.8 ^a^		25.3 ± 50.5 ^a^			758	754
(Z)-3-Hexenal	181.8 ± 226.3 ^c^	32.3 ± 78.9 ^a^	116.5 ± 115.7 ^abc^	117.7 ± 129.2 ^abc^		59.1 ± 167.2 ^ab^	89.6 ± 121.1 ^abc^	152.8 ± 198.8^bc^		14.4 ± 64.3 ^a^	800	798
Hexanal	3189.8 ± 1057.7 ^cd^	2376.5 ± 913.3 ^abc^	1716.4 ± 1281.4 ^ab^	1523.6 ± 822.2 ^ab^	2451.1 ± 1281.2 ^bc^	1441.9 ± 1515.3 ^ab^	3774.7 ± 2983.8 ^d^	1457.7 ± 973.8 ^ab^	1101.3 ± 1008.3 ^a^	2539.4 ± 938.2 ^bc^	803	798
(E)-2-Hexenal	27,638.6 ± 4366.2 ^c^	14,650.8 ± 6449.5 ^b^	8189.5 ± 5980.5 ^a^	15,450.5 ± 11,180.0 ^b^	2905.4 ± 1912.1 ^a^	5506.3 ± 3055.5 ^a^	19,133.1 ± 11,778.4 ^b^	17,274.3 ± 9844.7 ^b^	4486.1 ± 2568.5 ^a^	7010.0 ± 5588.7 ^a^	858	852
Heptanal	64.5 ± 52.6 ^a^	45.2 ± 59.9 ^a^	43.0 ± 121.7 ^a^	10.6 ± 33.5 ^a^	57.8 ± 75.5 ^a^	20.2 ± 43.4 ^a^	270.2 ± 382.0 ^b^	60.2 ± 134.6 ^a^		140.1 ± 113.2 ^a^	904	899
(E,E)-2,4-Hexadienal	323.0 ± 106.4 ^b^	76.7 ± 123.9 ^a^	102.6 ± 141.9 ^a^	85.4 ± 175.4 ^a^		8.5 ± 15.9 ^a^	92.0 ± 133.0 ^a^	272.7 ± 247.0^b^			916	916
(E)-2-Heptenal	139.2 ± 111.2 ^b^	81.6 ± 78.3 ^ab^	27.3 ± 77.3 ^ab^	61.5 ± 108.6 ^ab^	58.5 ± 90.1 ^ab^		261.4 ± 241.0 ^c^	53.6 ± 78.4 ^ab^		93.0 ± 136.8 ^ab^	961	963
Benzaldehyde	24.7 ± 40.6 ^ab^				3.0 ± 11.7 ^a^		30.5 ± 77.4 ^ab^	62.5 ± 102.9 ^b^		4.7 ± 20.4 ^a^	974	970
(E,E)-2,4-Heptadienal	364.8 ± 514.6 ^ab^	207.1 ± 292.3 ^ab^	377.5 ± 162.3 ^ab^	394.9 ± 183.9 ^ab^	91.1 ± 131.2 ^a^	289.0 ± 206.9 ^ab^	161.9 ± 113.3 ^ab^	457.4 ± 203.7 ^b^	177.0 ± 64.4 ^ab^	144.5 ± 137.4 ^a^	1002	1008
Octanal		20.7 ± 51.8 ^a^	144.2 ± 318.0 ^a^		75.5 ± 154.7 ^a^		83.3 ± 175.5 ^a^			100.5 ± 120.8 ^a^	1006	1004
Nonanal	334.3 ± 120.1 ^a^	291.5 ± 168.2 ^a^	244.3 ± 277.6 ^a^	123.0 ± 138.8 ^a^	435.1 ± 438.2 ^a^	246.9 ± 338.2 ^a^	1203.8 ± 1528.3 ^a^	330.3 ± 469.5 ^a^	66.2 ± 74.9 ^a^	1028.2 ± 699.5 ^b^	1108	1099
**Total Aldehydes**	**32552.7**	**17937.7**	**11030.2**	**19447.0**	**6293.1**	**7642.5**	**25439.9**	**20277.7**	**5838.4**	**11889. 9**		
**Ketones**												
2-Propanone	1437.9 ± 1814.9 ^ab^			3406.4 ± 3664.4 ^b^	1781.0 ± 1682.8 ^ab^	2409.3 ± 720.9 ^ab^	3199.5 ± 1683.8 ^b^	342.3 ± 815.7 ^a^	356.2 ± 388.9 ^a^	2049.5 ± 2950.5 ^ab^	<500	-
1-Penten-3-one	284.3 ± 148.8 ^b^	9.1 ± 22.4 ^a^	232.1 ± 232.3^b^	68.1 ± 126.0 ^a^	27.7 ± 63.4 ^a^	69.6 ± 118.6 ^a^	41.3 ± 57.6 ^a^	262.2 ± 252.4^b^	26.4 ± 64.7 ^a^	75.0 ± 100.4 ^a^	685	678
2-Pentanone		61.8 ± 222.8 ^a^			31.2 ± 60.0 ^a^	69.0 ± 130.0 ^a^	10.5 ± 33.3 ^a^			46.5 ± 109.2 ^a^	686	689
3-Pentanone	105.7 ± 76.8 ^abc^	69.9 ± 64.2 ^ab^	144.1 ± 111.0 ^abc^	117.5 ± 109.2 ^abc^	103.7 ± 62.7 ^abc^	410.4 ± 271.3^d^	38.7 ± 77.5 ^a^	217.3 ± 190.2 ^c^	189.5 ± 65.9 ^bc^	60.2 ± 58.8 ^ab^	696	694
2-Heptanone			10.6 ± 30.1 ^a^		264.9 ± 559.7 ^b^		14.3 ± 39.8 ^a^			1.6 ± 9.8 ^a^	891	889
6-Methyl-5-hepten-2-one	130.0 ± 91.6 ^abc^	65.2 ± 84.6 ^a^	84.0 ± 107.1 ^a^	38.1 ± 66.9 ^a^	260.6 ± 240.3 ^c^	47.8 ± 57.1 ^a^	245.7 ± 350.8 ^bc^	112.7 ± 183.2 ^ab^		57.6 ± 86.2 ^a^	986	985
2-Octanone					275.9 ± 588.4 ^b^		20.3 ± 49.9 ^a^			12.4 ± 30.1 ^a^	992	989
**Total Ketones**	**1957.8**	**206.0**	**470.8**	**3630.0**	**2745.2**	**3006.7**	**3570.2**	**934.4**	**572.2**	**2302.9**		
**Esters**												
(Z)-3-Hexenyl acetate	425.0 ± 333.9 ^a^	258.8 ± 261.4 ^a^			262.9 ± 298.5 ^a^	2698.5 ± 1110.6 ^c^	311.0 ± 431.4 ^a^	1986.2 ± 1224.8 ^b^	2021.0 ± 1291.3 ^b^	232.4 ± 390.8 ^a^	1004	1005
Hexyl acetate	93.9 ± 73.7 ^a^	93.7 ± 87.1 ^a^			76.5 ± 104.4 ^a^	608.6 ± 307.8 ^bc^	69.3 ± 142.1 ^a^	724.3 ± 393.5 ^bc^	849.7 ± 367.2 ^c^	61.1 ± 131.3 ^a^	1010	1011
**Total Esters**	**518.9**	**352.4**			**339.4**	**3307.1**	**380.3**	**2710.4**	**2870.6**	**293.5**		
**Terpenes**												
α-Pinene	57.7 ± 135.4 ^a^	29.1 ± 104.8 ^a^		31.2 ± 99.9 ^a^	3997.5 ± 5975.8 ^b^	154.6 ± 300.7 ^a^	654.6 ± 1452.6 ^a^	53.2 ± 195.0 ^a^	28.8 ± 44.9 ^a^		945	932
δ-3-Carene		6.8 ± 24.4 ^a^			130.6 ± 273.6 ^b^		133.0 ± 310.1 ^b^				1022	1010
p-Cymene	37.3 ± 66.6 ^a^	50.1 ± 64.9 ^a^		4.4 ± 20.1 ^a^	49.0 ± 88.8 ^a^	176.4 ± 407.6 ^b^	47.9 ± 63.1 ^a^	9.1 ± 28.1 ^a^		36.4 ± 59.0 ^a^	1035	1023
dl-Limonene	39.0 ± 55.2 ^a^	874.0 ± 3061.2 ^a^	27.1 ± 50.2 ^a^	63.6 ± 72.0 ^a^	420.0 ± 1095.6 ^a^	3515.7 ± 5393.3 ^b^	665.9 ± 1757.8 ^a^	76.3 ± 224.9 ^a^		87.6 ± 326.7 ^a^	1041	1035
(E)-β-Ocimene	258.5 ± 77.9 ^a^	157.0 ± 201.4 ^a^	411.6 ± 299.2 ^a^	310.1 ± 286.0 ^a^	262.8 ± 136.4 ^a^	172.9 ± 148.7 ^a^	2009.3 ± 2244.1 ^b^	179.4 ± 315.4 ^a^	18.8 ± 29.4 ^a^	13.4 ± 33.5 ^a^	1049	1035
γ-Terpinene	0.9 ± 3.9 ^a^				16.9 ± 65.5 ^a^	306.6 ± 729.1 ^b^	44.2 ± 174.3 ^a^			2.0 ± 12.0 ^a^	1068	1048
allo-Ocimene					7.5 ± 29.1 ^a^		47.5 ± 87.6 ^b^				1146	1129
α-Copaene	182.7 ± 47.2 ^b^	101.9 ± 87.9 ^ab^	748.0 ± 309.4^c^	197.4 ± 193.2 ^b^	31.1 ± 40.7 ^a^	16.6 ± 2.9 ^a^	4.4 ± 20.2 ^a^	3.8 ± 16.2 ^a^	37.7 ± 70.2 ^a^	1.1 ± 6.6 ^a^	1404	1392
(E)-β-Farnesene					31.4 ± 46.7 ^b^						1467	1471
(E,E)-α-Farnesene	282.8 ± 221.8 ^bcd^	325.8 ± 278.9 ^cd^	142.8 ± 157.7 ^abc^	194.6 ± 257.2 ^abc^	518.4 ± 371.7 ^d^	62.5 ± 67.9 ^ab^	151.9 ± 135.6 ^abc^	18.5 ± 45.1 ^a^			1512	1509
α-Muurolene	6.7 ± 30.7 ^a^		72.9 ± 80.3 ^b^	20.2 ± 44.4 ^a^	11.2 ± 43.4 ^a^		22.6 ± 73.7 ^a^	7.3 ± 31.0 ^a^			1526	1530
**Total Terpens**	**865.5**	**1544.7**	**1402.3**	**821.5**	**5476.3**	**4405.3**	**3781.4**	**347.6**	**85.4**	**140.5**		
**Hydrocarbons**												
1,3-Butadiene, 2-methyl-	35.2 ± 64.0^b^							13.4 ± 43.1 ^a^			<500	-
(Z)-1,3-Pentadiene	26.4 ± 36.7 ^a^			5.6 ± 25.7 ^a^		17.2 ± 48.7 ^a^		56.3 ± 81.5^b^			<500	-
Pentane, 2,2,4-trimethyl-	1335.7 ± 3943.5 ^a^	4438.7 ± 5333.5 ^a^	1078.9 ± 1220.1 ^a^	14,039.8 ± 12,531.5 ^b^	22.9 ± 61.2 ^a^		10,369.6 ± 13,701.5 ^b^	639.9 ± 1204.6 ^a^	149.1 ± 73.4 ^a^	510.7 ± 955.7 ^a^	689	668
Heptane	80.7 ± 121.1 ^a^			236.4 ± 595.5 ^a^	198.4 ± 246.2 ^a^	87.2 ± 161.4 ^a^	165.7 ± 327.8 ^a^	23.8 ± 56.3 ^a^		180.1 ± 289.8 ^a^	700	700
Toluene	69.9 ± 80.4 ^a^			15.6 ± 50.8 ^a^	10.3 ± 21.8 ^a^	106.8 ± 119.6 ^a^	256.8 ± 774.6 ^a^	212.3 ± 295.1 ^a^	12.6 ± 31.0 ^a^	10.3 ± 29.9 ^a^	773	771
1-Octene		27.3 ± 34.2 ^a^			39.2 ± 69.1 ^a^		68.4 ± 143.3 ^a^			70.5 ± 110.4 ^a^	793	792
2-Octene		17.9 ± 64.6 ^a^			7.0 ± 26.9 ^a^		150.2 ± 313.4 ^b^			22.0 ± 75.5 ^a^	818	815
Xylene	34.8 ± 47.4 ^a^			5.1 ± 16.0 ^a^	21.1 ± 38.4 ^a^	92.6 ± 111.1 ^a^	311.6 ± 1013.2 ^a^	259.9 ± 581.8 ^a^		9.5 ± 21.8 ^a^	879	870
Cyclopentane, 2-propenyl-	235.7 ± 91.2 ^c^	76.9 ± 53.5 ^ab^	108.7 ± 48.9 ^b^	49.2 ± 53.7 ^ab^	30.7 ± 42.6^b^	76.5 ± 49.8 ^ab^	48.7 ± 58.9 ^ab^	111.6 ± 42.4 ^b^	21.6 ± 34.2 ^b^	17.4 ± 34.4 ^a^	898	850
Styrene		22.3 ± 43.8 ^a^			57.5 ± 158.2 ^ab^		165.5 ± 373.3 ^b^			39.5 ± 74.5 ^a^	901	895
Nonane		6.8 ± 24.6 ^a^			130.8 ± 211.1 ^b^						900	900
3-Ethyl-1,5-octadiene	858.9 ± 343.2 ^c^	430.9 ± 232.7 ^ab^	618.6 ± 160.0 ^bc^	379.2 ± 184.6 ^ab^	214.2 ± 175.7 ^a^	412.3 ± 200.2 ^ab^	265.4 ± 195.5 ^a^	595.4 ± 247.4 ^bc^	283.8 ± 84.7 ^a^	198.7 ± 148.4 ^a^	939	949
Benzene, 1,3,5-trimethyl-		27.9 ± 27.8 ^a^					53.9 ± 208.9 ^a^				978	976
1,7-Nonadiene, 4,8-dimethyl	569.4 ± 524.8 ^b^	330.7 ± 347.0 ^ab^	530.2 ± 121.4 ^b^	402.1 ± 170.3 ^ab^	139.4 ± 179.4 ^a^	345.5 ± 286.5 ^ab^	166.8 ± 221.3 ^a^	550.1 ± 273.4 ^b^	247.9 ± 78.7 ^ab^	154.3 ± 165.0 ^a^	998	1026
Undecane			186.5 ± 36.5 ^c^		118.3 ± 162.3 ^b^						1100	1100
(E)-4,8-Dimethyl-1,3,7-nonatriene	546.1 ± 208.7 ^c^	404.9 ± 431.0 ^bc^	57.1 ± 84.4 ^a^	19.3 ± 43.5 ^a^	10.1 ± 27.4 ^a^	50.3 ± 56.7 ^a^	385.9 ± 336.9 ^bc^	203.2 ± 158.5 ^ab^		193.8 ± 120.2 ^ab^	1116	-
Dodecane			120.1 ± 24.3 ^b^		12.5 ± 33.2 ^a^						1200	1200
Cyclodecane, methyl-	276.2 ± 189.3 ^a^	229.0 ± 288.6 ^a^	1368.3 ± 896.3 ^c^	814.3 ± 471.2 ^b^	3451.8 ± 1527.7 ^d^	162.8 ± 182.4 ^a^	598.0 ± 439.9 ^ab^	274.7 ± 349.7 ^a^	881.4 ± 483.7 ^b^	73.7 ± 302.1 ^a^	1208	1202
**Total Hydrocarbons**	**4069.1**	**6013.5**	**4068.4**	**15966.5**	**4464.2**	**1351.1**	**13006.4**	**2940.6**	**1596.5**	**1480.5**		
**Miscellaneous**												
Ethyl ether	87.4 ± 169.9 ^a^		11.1 ± 31.4 ^a^	89.9 ± 175.7 ^a^		43.6 ± 123.4 ^a^	76.7 ± 162.8 ^a^	39.2 ± 77.0 ^a^	27.7 ± 67.8 ^a^	97.6 ± 177.0 ^a^	<500	-
Hexane, 1-methoxy-	1120.3 ± 600.6 ^c^	867.9 ± 432.2 ^b^			4.1 ± 15.9 ^a^	192.3 ± 206.0 ^a^			56.4 ± 138.1 ^a^		831	832
(Z)-3-Hexene, 1-methoxy-	353.7 ± 342.8 ^b^	354.0 ± 283.0 ^b^			3.0 ± 11.6 ^a^	19.6 ± 55.5 ^a^					832	801
**Total Miscellaneous**	**1561.4**	**1221.8**	**11.1**	**89.9**	**7.1**	**255.6**	**76.7**	**39.2**	**84.0**	**97.6**		
**Total Volatiles**	**43,172.2**	**29,976.0**	**18,154.6**	**42,132.9**	**21,925.8**	**24,631.4**	**47,765.9**	**30,084.5**	**14,437.8**	**17,232.2**		

Means with different letters in the same row are significantly different (*p* < 0.05), 1: RI_lit_: literature retention index, 2: RI_exp_: experimental retention index (NIST MS search), 3: not calculated.

**Table 4:** Discriminant functions formed and Linear Discriminant Analysis (LDA) and Stepwise LDA (SLDA) results (eigenvalues, explained variance, canonical correlation, W’Lambda, X^2^, df and p for each function).

Discriminant Function	Eigenvalue	Variance %	Cumulative %	Can. Correlation	Wilks’ Lambda	X^2^	df	*p* < 0.05
**LDA**								
1	38.110	48.2	48.2	0.987	0.001	2146.769	495	0.000
2	15.276	19.3	67.6	0.969	0.001	1657.308	432	0.001
3	9.070	11.5	79.0	0.949	0.004	1284.882	371	0.001
**SLDA**								
1	10.900	33.8	33.8	0.957	0.001	1649.290	162	0.000
2	8.938	27.7	61.6	0.948	0.001	1272.862	136	0.000
3	4.778	14.8	76.4	0.909	0.002	923.820	112	0.000

**Table 5:** Variables-predictors produced from SLDA.

**Step**	Variables in the Analysis	F-Statistic	df1	df2	*p*
1	Dodecane	98.135	9	157.000	0.001
2	Hexane, 1-methoxy-	69.985	18	312.000	0.000
3	Cyclodecane, methyl-	60.438	27	453.322	0.001
4	Hexyl acetate	55.047	36	578.847	0.000
5	α-Copaene	46.783	45	687.509	0.001
6	Cyclopentane, 2-propenyl-	40.908	54	779.645	0.001
7	(*E*)-β-Ocimene	36.183	63	856.548	0.001
8	Nonanal	33.016	72	919.994	0.000
9	Ethanol	30.783	81	971.886	0.001
10	(*E,Ε*)-2,4-Heptadienal	29.134	90	1014.043	0.000
11	(*E*)-2-Hexenal	25.685	108	1075.417	0.001
12	(*E*)-2-Hexenol	24.204	117	1097.196	0.001
13	3-Pentanone	23.118	126	1114.392	0.000
14	dl-Limonene	22.118	135	1127.794	0.001
15	1-Penten-3-ol	21.362	144	1138.046	0.000
16	(*E*)-2-Pentenal	20.647	153	1145.675	0.001
17	2-Propanone	20.022	162	1151.106	0.001

At each step, the variable that minimizes the overall Wilks’ Lambda is entered. Maximum number of steps is 110. Minimum partial F to enter is 3.84. Maximum partial F to remove is 2.71.
